# Diffuse leptomeningeal glioneuronal tumor with high-grade features masquerading as tubercular meningitis—a case report

**DOI:** 10.1186/s43055-021-00522-0

**Published:** 2021-06-11

**Authors:** Sameer Peer, Vivek Murumkar, Karthik Kulanthaivelu, Chandrajit Prasad, Shilpa Rao, Vani Santosh

**Affiliations:** 1 Department of Neuroimaging and Interventional Radiology, National Institute of Mental Health and Neurosciences, Bengaluru, Karnataka 560029 India; 2 Department of Neuropathology, National Institute of Mental Health and Neurosciences, Bengaluru, Karnataka 560029 India

**Keywords:** Diffuse leptomeningeal glioneuronal tumor, Enhancement, Anaplasia, Meningitis, Case report

## Abstract

**Background:**

Diffuse leptomeningeal glioneuronal tumor (DLGNT) has been recently described in the literature. The complete neuroimaging spectrum and histopathological characteristics of this entity are yet to be elucidated. In an endemic region, diffuse leptomeningeal enhancement on neuroimaging with associated communicating hydrocephalus is usually suggestive of infective meningitis and the patients are started on empirical anti-microbial therapy. However, it is important to consider other differential diagnosis of leptomeningeal enhancement in such cases, particularly if the clinical condition does not improve on anti-microbial therapy. An early diagnosis of a neoplastic etiology may be of particular importance as the treatment regimens vary considerably depending on the underlying disease condition.

**Case presentation:**

In this case report, we describe a case of DLGNT with high-grade histopathological features which was initially managed as tubercular meningitis based on the initial neuroimaging findings. Due to worsening of the clinical course and subsequent imaging findings at follow-up, a diagnosis of DLGNT was considered and subsequently proven to be DLGNT with features of anaplasia on histopathological examination of leptomeningeal biopsy specimen.

**Conclusion:**

This case highlights the importance of recognizing certain subtle finding on MRI which may help in an early diagnosis of DLGNT which is crucial for appropriate treatment.

## Background

Leptomeningeal enhancement (LME), also known as Pia-Arachnoid enhancement, is a pattern of enhancement that involves the subarachnoid spaces [1]. The implicated etiologies include infection (bacterial, viral, or fungal meningitis), inflammation (sarcoidosis), or neoplasms [1]. Among neoplasms, the leptomeningeal extension may emanate from primary CNS neoplasms (in cases of medulloblastoma, ependymoma, pineoblastoma, germinoma, high-grade gliomas) or secondary leptomeningeal metastasis from tumors such as the breast, lung, ovarian malignancies, lymphoproliferative disorders, or melanoma [1, 2]. Diffuse Leptomeningeal glioneuronal tumor (DLGNT), a recently characterized neoplastic entity, exhibits the characteristic imaging appearance of diffuse leptomeningeal enhancement and multiple sub-pial cysts [3]. Hydrocephalus may be the initial presentation [4]. The imaging appearance and indolent clinical features may lead to a misdiagnosis of the cases of DLGNT as chronic meningitis [5]. We illustrate a case of raised intracranial tension due to communicating hydrocephalus, where the imaging features simulated DLGNT (in its pattern of leptomeningeal spread) with histomorphological examination revealing features of a high-grade glioneuronal tumor, favoring the diagnosis of DLGNT with histological features of anaplasia.

## Case presentation

**Fig. 1:**
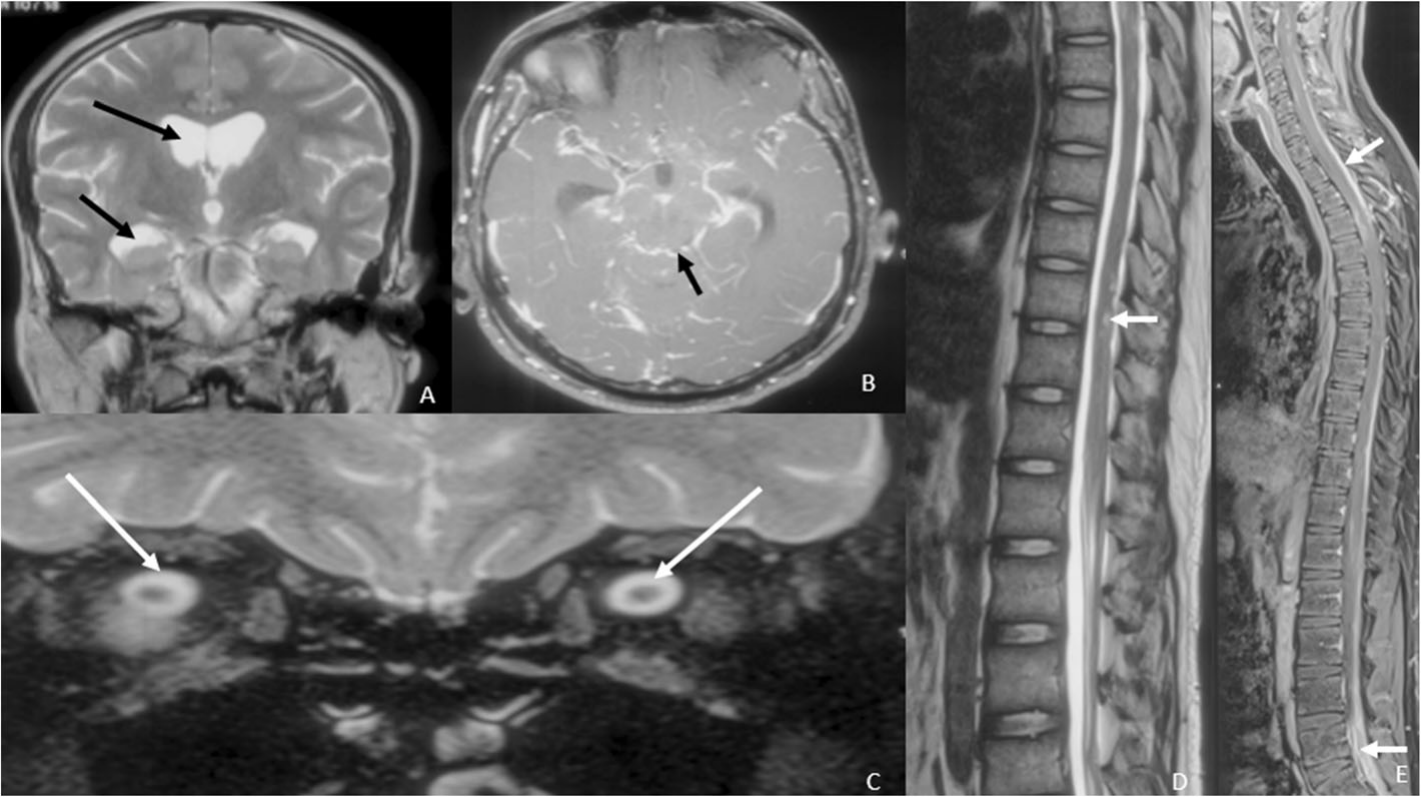
Magnetic resonance imaging of the brain and dorsal spine at 2 months following the onset of symptoms. **A** T2W coronal image of the brain at the level of the foramen of Monro shows dilated bilateral lateral ventricles. The black arrows point to the dilated frontal and temporal horn of the right lateral ventricle. **B** T1W post-contrast image of the brain at the level of the mid-brain shows diffuse and thin leptomeningeal enhancement. The black arrow is pointing towards the leptomeningeal enhancement along the quadrigeminal cistern. **C** T2W coronal image of the brain at the level of the orbits (zoomed to demonstrate optic nerves) shows distended per-optic CSF spaces. The white arrows point towards the distended peri-optic CSF spaces. **D** Sagittal T2W MRI of the dorsal spine shows few cysts along the dorsal spinal cord. The white arrows point towards the intramedullary cysts within the dorsal cord. **E** Post-contrast T1W sagittal image of the spine. There is diffuse leptomeningeal enhancement noted along the cord surface and the nerve roots of cauda equina. The white arrows point towards the leptomeningeal enhancement along the dorsal cord and also the cauda equina

**Fig. 2:**
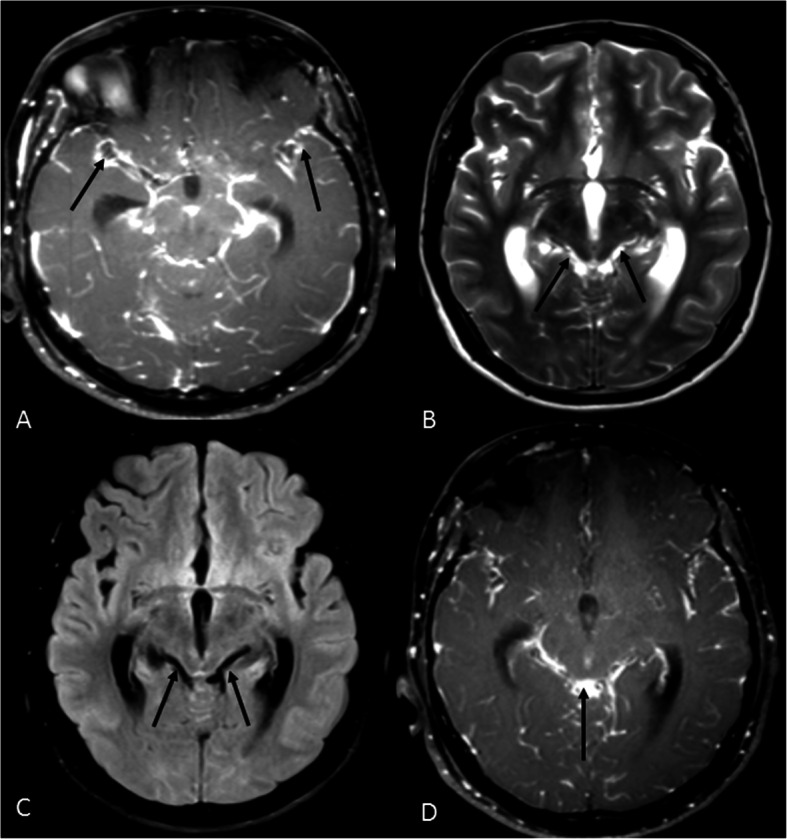
Magnetic resonance imaging of the brain at 2 months following the onset of symptoms. **A** Axial T1W post-gadolinium image of the brain at the level of the mid-brain shows nodularity of leptomeningeal enhancement. The black arrows point towards the nodular leptomeningeal enhancement along the Sylvian fissures. **B** T2W axial image of the brain at the level of the mid-brain shows expansion of the quadrigeminal cistern with inhomogeneous signal of the CSF. The black arrows are seen to point towards the inhomogeneous CSF signal in the quadrigeminal cistern. **C** Axial FLAIR image of the brain at the level of mid-brain shows complete signal suppression as compared with T2W image in **B**. The black arrows depict the suppression of CSF signal on FLAIR. **D** Post-contrast T1W axial images of the brain show enhancement along the CSF spaces corresponding to the T2 and FLAIR images. The black arrows point towards the enhancement along the CSF spaces

**Fig. 3:**
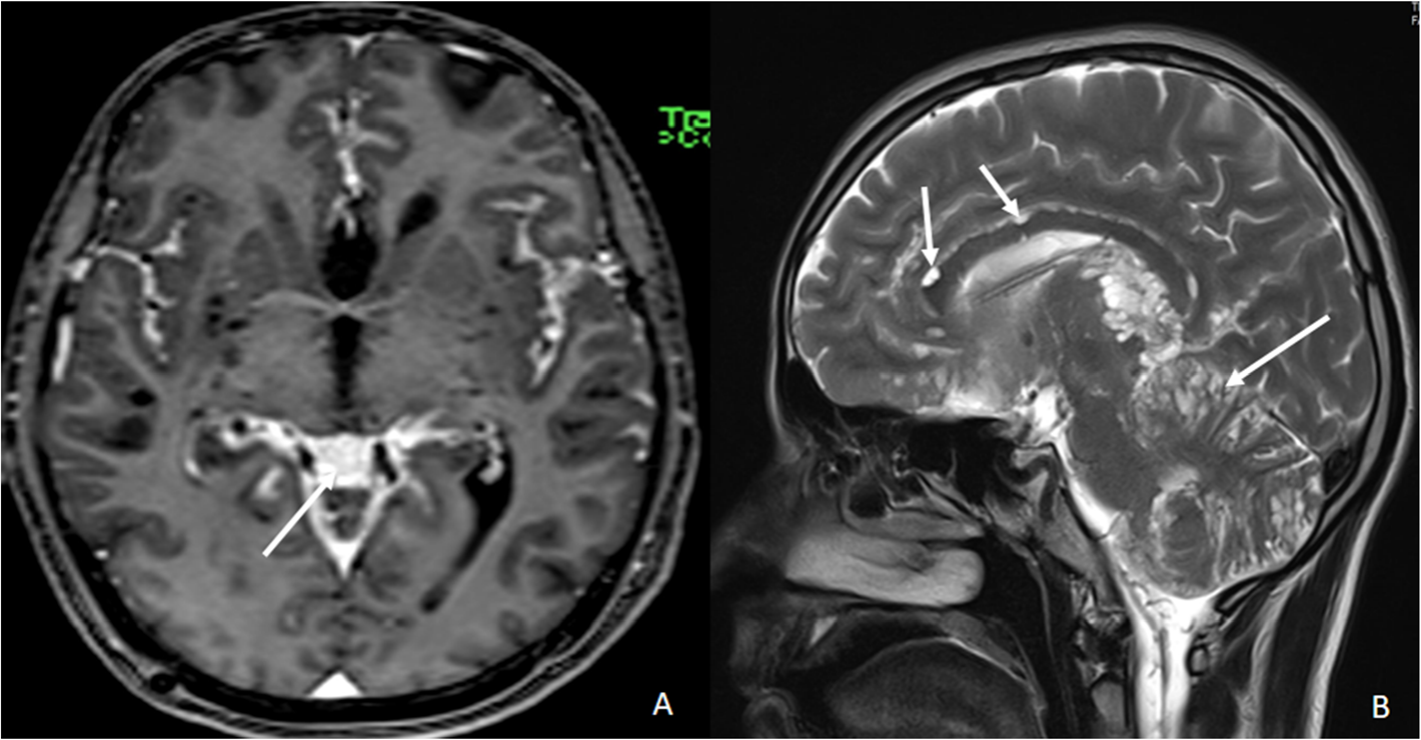
Follow-up magnetic resonance imaging of the brain at our institute 8 months after the onset of the symptoms. **A** Post-contrast T1W axial image of the brain at the basal ganglia level shows enhancement along the CSF spaces. The white arrow points towards the enhancement along the CSF spaces in the velum interpositum cistern. **B** T2W sagittal image of the brain in the mid-sagittal plane shows multiple small cysts of varying sizes along the cerebellar folia and the pial surfaces of the corpus callosum. The white arrows point towards the small cysts along the cerebellar folia and the corpus callosum

**Fig. 4:**
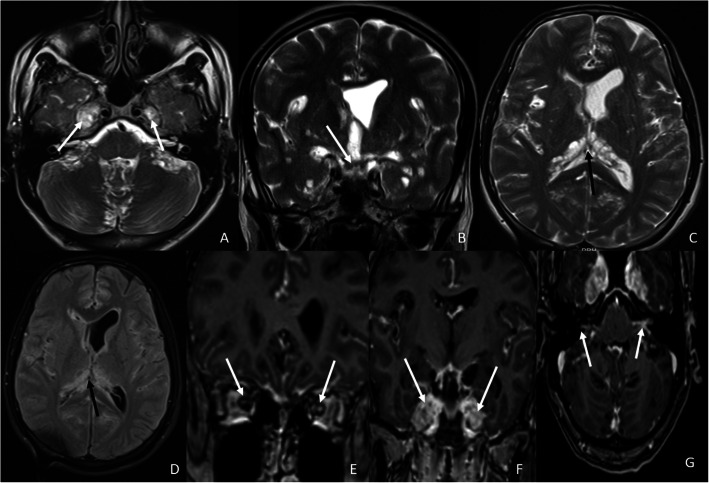
Follow-up magnetic resonance imaging of the brain at our institute 8 months after the onset of the symptoms. **A** T2W axial image of the brain at the level of the Meckel’s caves shows ballooning of the Meckel’s cave bilaterally. The white arrows point towards the ballooning of bilateral Mecksl’s caves. **B** T2W coronal image of the brain at the level of the optic chiasm shows expanded chiasmatic cistern with displaced optic chiasm. The white arrow points towards the displaced optic chiasm. **C** T2W axial image of the brain at the level of the velum interpositum. There is expansion of the velum interpositum with hyperintense signal as demonstrated with black arrow. **D** Axial FLAIR image of the brain at the level of velum interpositum shows no signal suppression in the velum intrepositum on FLAIR, as pointed out with black arrow, in comparison with T2W image shown in **C**. **E** Post-contrast T1W coronal image of the brain at the level of the orbits depicts enhancement along bilateral optic nerves as pointed out with white arrows. **F** Post-contrast T1W coronal image at the level of the Meckel’s cave shows avid enhancement is noted along bilateral Meckel’s caves and internal acoustic canals as shown with the help of white arrows. **G** Post-contrast T1W axial image at the level of the internal acoustic canals showing avid contrast enhancement along the bilateral Meckel’s caves and the internal acoustic canals as demonastrated with white arrows

**Fig. 5:**
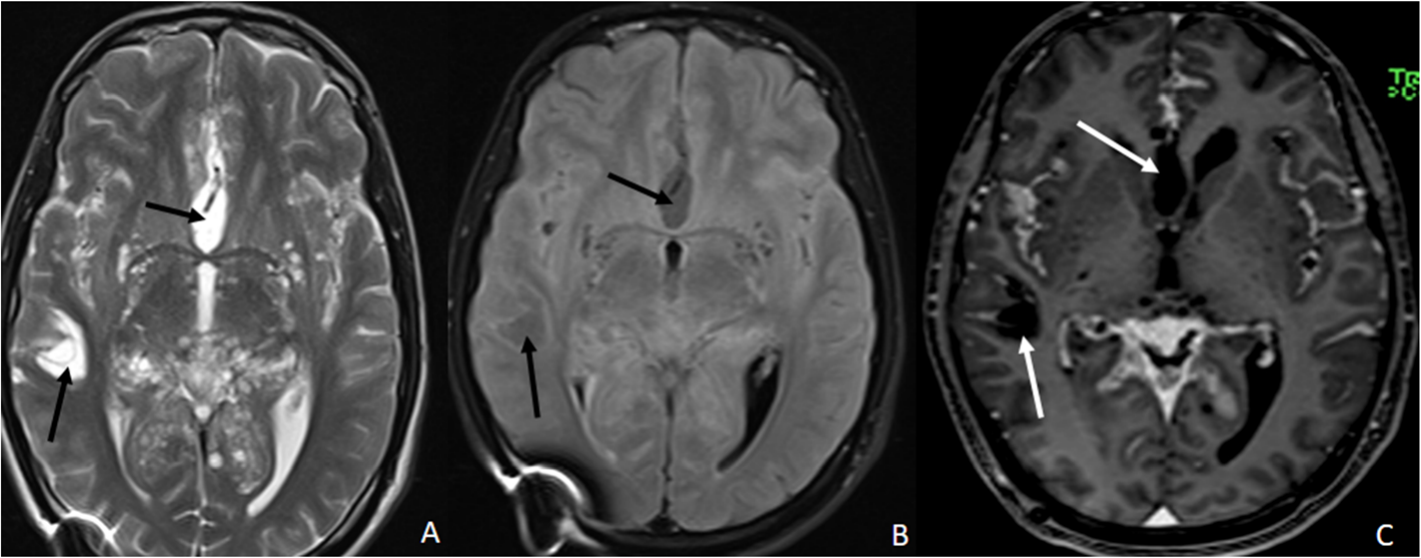
Follow-up magnetic resonance imaging of the brain at our institute 8 months after the onset of the symptoms. **A** T2W axial image of the brain at the level of the basal ganglia shows T2 hyperintense cystic structures in the right temporal operculum and the anterior interhemispheric fissure as pointed out with the help of black arrows. **B** Axial FLAIR image of the brain at the level of the basal ganglia shows the signal suppression in the cystic lesions on FLAIR as compared with T2W image shown in **A**. This finding is pointed out with the black arrows. **C** Post-contrast T1W axial image of the brain at the level of basal ganglia shows no evidence of enhancement of the cystic structures, pointed out with white arrows. These cystic structures may represent encysted or loculated CSF

A 21-year-old male presented at our institute with complaints of holocranial headache, vomiting, and blurring of vision of an 8-month duration. Neuroimaging evaluation performed elsewhere at 2 months following the onset of symptoms showed communicating hydrocephalus and features of raised intracranial tension (bilateral peri-optic CSF space prominence). Diffuse thin leptomeningeal enhancement was seen enveloping the entire neuraxis. Few small cysts were noted along the dorsal spinal cord (Fig. 1). With an empirical diagnosis of tubercular meningitis with hydrocephalus (TBM with HCP), anti-tubercular therapy was initiated empirically and ventriculo-peritoneal shunt was placed. The patient received an oral fixed dose combination of isoniazid (75 mg), rifampicin (150 mg), pyrazinamide (400 mg), and ethambutol (275 mg) and was administered as 3 tablets per day for 2 months. This was followed by an oral fixed dose combination of isoniazid (75 mg), rifampicin (150 mg), and ethambutol (275 mg), given as 3 tablets per day for 4 months. The patient tolerated the anti-tubercular medication without any adverse effects. However, the patient clinically deteriorated over the next 6 months despite antitubercular therapy and ventriculoperitoneal shunt placement. A review of the imaging acquired elsewhere at the first time point (2 months) was done at our institute. Nodularity of enhancement was observed at a few foci. The basal cisterns and bilateral Meckel’s caves appeared prominent with inhomogeneous CSF signal on T2W images. In spite of complete suppression of the signal on FLAIR in the abovementioned areas, an intense post-contrast enhancement was observed in the corresponding regions (Fig. 2). An MRI performed at the current presentation showed an interval increase in the thickness and extent of the leptomeningeal enhancement. Multiple discrete, well-defined sub-pial cysts were present along the bilateral cerebral hemispheres, corpus callosum, cerebellum, brainstem, and along the spinal cord (Fig. 3). On follow-up MRI, an extension of the lesion was evident with more florid involvement of the basal cisterns and ballooning of the Meckel’s caves was noted bilaterally. The lesion was distorting the optic chiasm and appeared to extend along the left optic nerve sheath. However, there was an absence of lesional FLAIR inversion in the current scan, and more avid enhancement was evident. Asymmetric enhancement (left > right) was also noted along the bilateral optic nerves (Fig. 4), Meckel’s cave, and internal acoustic canal. Within the sulcal spaces, the admix of CSF loculations and tumoral cyst were evident, weighing in the non-enhancement, FLAIR inversion possibly corresponded to loculated CSF (Fig. 5). Susceptibility and diffusion characteristics were unremarkable. Considering the distinct imaging findings, a diagnosis of DLGNT was suggested. The CSF analysis, with 30 ml of sample volume obtained with standard lumbar puncture technique, showed 24 cells/mm^3^ (lymphocytes) with no atypical cells. Previous CSF analysis report was not available with the patient for comparison. Nucleic acid amplification test was negative for *Mycobacterium tuberculosis*. The CSF protein level was 3174 mg/dl (normal range 15–45 mg/dl), CSF glucose level was 104 mg/dl (normal range 40–0 mg/dl), and CSF lactate level was 38 mg/dl (normal range 10–22 mg/dl). CSF culture failed to grow any organism at 48 h. India Ink staining for *Cryptococcus neoformans*, fungal stains, and fungal cultures were negative.

**Fig. 6:**
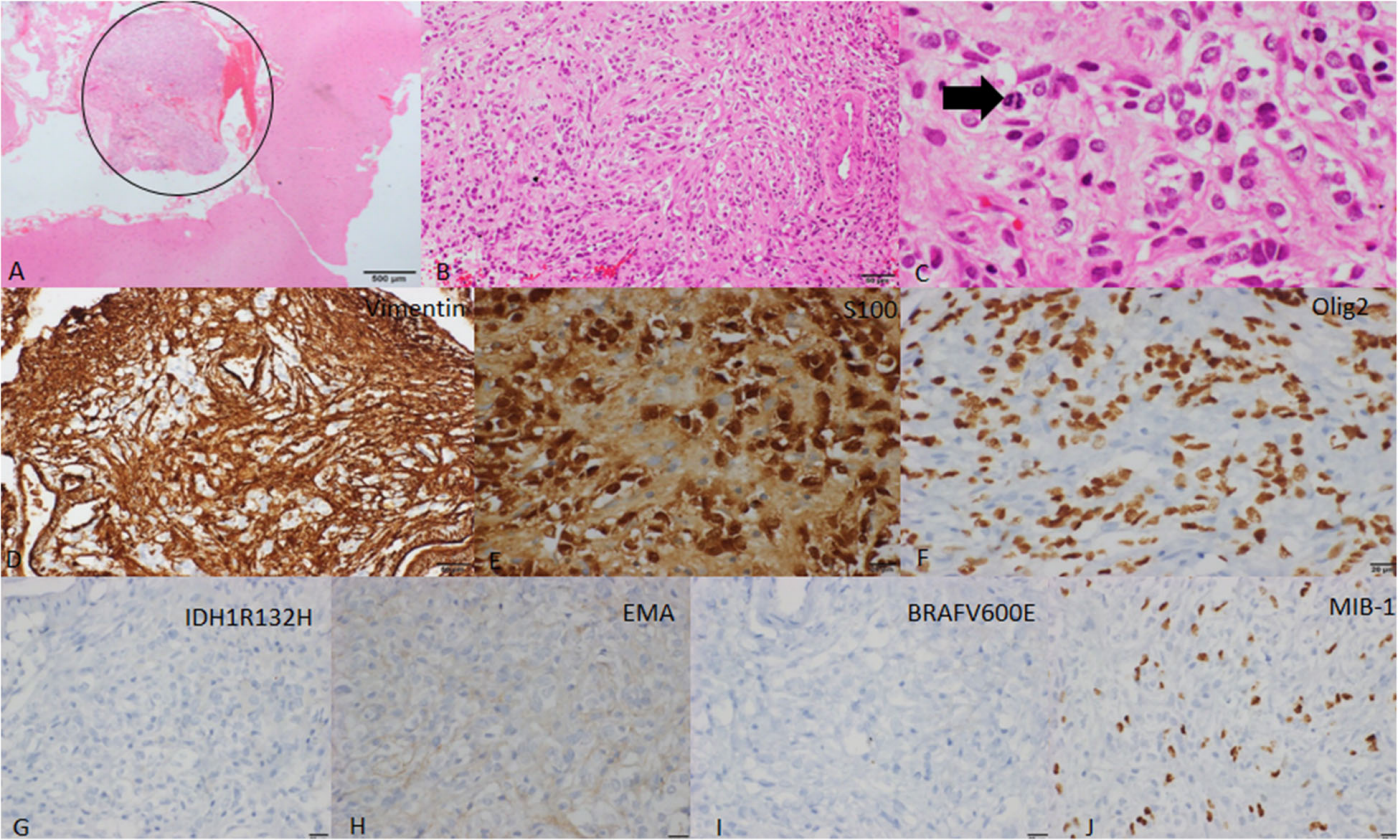
Histopathological features of the leptomeningeal biopsy specimen. **A** Hematoxylin & eosin stain photomicrograph of the leptomeningeal biopsy specimen shows a small nodule of tumor within the tumor as shown with a circle. **B** Hematoxylin & eosin stain photomicrograph of the tumor region shows glial cells with eosinophilic clear cytoplasm. **C** Hematoxylin & eosin stain of the tumor shows mitotic figures within the glial cells as depicted with the help of the arrow. **D** Photomicrograph of the tumor with immunoperoxidase shows positivity for vimentin. **E** Photomicrograph of the tumor with immunoperoxidase shows positivity for S-100. **F** Photomicrograph of the tumor with immunoperoxidase shows positivity for Olig2. **G** Photomicrograph of the tumor with immunoperoxidase shows immunonegative response for IDH1R132H. **H** Photomicrograph of the tumor with immunoperoxidase shows immunonegative reaction for EMA. **I** Photomicrograph of the tumor with immunoperoxidase shows immunonegativity for BRAFV600E. **J** Photomicrograph of the tumor with immunoperoxidase shows moderate proliferation of the tumor cells (MIB-1)

Since routine diagnostic workup was inconclusive with regard to the etiology, right temporal biopsy was performed in view of the deteriorating clinical condition and concomitant imaging findings. The sampled tissue revealed a small nodule of tumor cells within the leptomeninges, which was composed of cells exhibiting oligodendrocyte-like cytological features exhibiting increased mitotic activity. These cells were immunonegative for GFAP; however, they displayed immunopositivity for Olig2 and S-100. Immunomarkers for diffuse glioma (IDH1R132H, ATRX, p53) and ependymoma (EMA) were negative (Fig. 6). Histological and immunohistochemical features were of a high-grade diffuse glioneuronal tumor. The antitubercular treatment was discontinued, and the patient was advised to see an oncology center for further management.

At 3 months’ follow-up, persistent headaches and intermittent vomiting continued unabated. The patient did not receive any chemotherapy or radiation therapy due to financial constraints, and the situation was worsened by imposition of lock-down in our country in view of the COVID-19 pandemic. As per the latest follow-up, the patient has relentless worsening of headache. The patient is bed bound and not able to carry out routine activities.

## Discussion

Gardiman et al. first described DLGNT in 2010 in four children who demonstrated thickened and enhancing subarachnoid spaces, more marked in the basal cisterns, and interhemispheric fissures with disseminated cystic lesions throughout the brain [6]. DLGNT usually afflicts children and young adults, and Rodriguez et al. reported the median age at 5 years (range between 5 months and 46 years) [7]. The common manifestations include headache, altered mental status, behavioral abnormalities, paraphasia, and hydrocephalus [4].

Neuroimaging of DLGNT has hitherto been considered characteristic of the entity. Leptomeningeal thickening and enhancement, along with multiple discrete sub-pial cysts involving the cerebrum, brainstem, and spinal cord, have been reported [3]. These cysts or T2-hyperintense foci may result from fibrosis and expansion of the subarachnoid spaces [3, 7]. Nodular enhancement along the brain and spinal cord surface and sugar-coat enhancement of the nerve roots are the other imaging attributes on T1 post-contrast imaging [7]. As in our case, intramedullary lesions are another feature of DLGNT [3, 7]. In the index case, the initial imaging was remarkable for hydrocephalus with diffuse leptomeningeal enhancement, and tubercular etiology was erroneously assigned. Importantly, on follow-up imaging, these changes were more widespread. The perioptic contrast enhancement was observed additionally. However, on a careful re-evaluation of the initial MRI, soft imaging markers emerged, which favored neoplastic etiology, namely, expansion of the basal cisterns and the Meckel’s caves with nonhomogeneous CSF signal on T2-weighted imaging and corresponding enhancement on post-contrast imaging. We intend to highlight the importance of these findings, which may help differentiate DLGNT from infective meningitis. The remarkable discordance between the normal FLAIR imaging (complete inversion) vis-a-vis a brilliant contrast enhancement of the CSF space argues against an exudative infective etiology. In the index case, initial time point MRI showed expanded CSF spaces in the basal cisterns and Meckel’s caves, which were hyperintense on T2W images and inverted on FLAIR. There was contrast enhancement along the expanded CSF spaces. Follow-up MRI did show avid contrast enhancement of the CSF spaces, but the CSF signal were not completely supressed on FLAIR imaging. Progressive accumulation of the neoplastic cells with marked elevation of the CSF proteins may account for the imaging evolution.

The recent WHO update (2016) on CNS tumors classified DLGNT as a distinct entity [3, 7]. On histopathology, these lesions show morphological features resembling oligodendroglioma, often with neurocytic/neuronal differentiation and a low mitotic index [6, 7]. On immunohistochemistry, the tumor cells show positivity for OLIG2, MAP2, S100, and less than 50% are immunopositive for GFAP. Synaptophysin, chromogranin A, and other neuronal markers can be positive in cases with overt neuronal features [7]. IDH1 (R132H) and EMA are negative. The characteristic molecular alteration of this tumor is concurrent KIAA1549-BRAF gene fusions and either solitary 1p deletion or 1p/19q codeletion in the absence of IDH mutation [7]. Recently, DNA methylation profiling has identified two molecular subgroups, MC-1 associated with a better prognosis, and MC-2, associated with a worse prognosis [8].

There is a broad differential diagnosis for diffuse leptomeningeal enhancement. The most common cause of diffuse leptomeningeal enhancement is meningitis, etiologically diverse. Among all the causes, bacterial and viral meningitis are the most common, exhibiting thin-linear meningeal enhancement [1]. Fungal meningitis exhibits thick and nodular enhancement, but the entire neuraxis being plastered with such pathology is unusual, besides the CSF picture is also incongruent. The neoplastic meningeal seeding or “carcinomatous meningitis” often demonstrate thick and nodular enhancement, a highly variable imaging finding [1]. Medulloblastoma and other CNS embryonal tumors, ependymoma, germinoma, pineoblastoma, and high-grade glioma commonly engender carcinomatous meningitis [1]. Involvement of the meninges by secondaries is commonly associated with malignancies originating from the lung and breast, lymphoproliferative disorders, and melanoma [1]. Moyamoya disease seldom show diffuse leptomeningeal enhancement [9]. Other entities exhibiting diffuse leptomeningeal enhancement include primary diffuse leptomeningeal gliomatosis and diffuse leptomeningeal glioneuronal tumor [6, 10]. We underscore the need for suspicion of DLGNT/DLGNT-like neoplasms in such cases demonstrating thick and nodular meningeal enhancement, as it has a bearing on the treatment approaches. While ATT forms the bedrock of TBM management, DLGNT is managed with chemotherapy and/or radiotherapy [4, 7]. Although slow-growing, secondary hydrocephalus leads to considerable morbidity, and the prognosis is hard to ascribe.

## Conclusion

In an endemic region, thick nodular meningeal enhancement with hydrocephalus evokes a diagnosis of TBM, which is remarkably similar to DLGNT, as highlighted by our case. The identification of sub-pial cysts may serve as an important imaging marker for the diagnosis of this entity. Also, subtle imaging clues, i.e., expanded, inhomogeneous appearing CSF spaces in conjunction with distinct enhancement, could help in the early diagnosis.

## Data Availability

The datasets used and/or analyzed during the current study are available from the corresponding author on reasonable request.

## Abbreviations

ATT: Anti-tubercular therapy
CNS: Central nervous system
CSF: Cerebrospinal fluid
DNET: Dysembroplastic neuroepithelial tumour
DLGNT: Diffuse leptomeningeal glioneuronal tumour
EMA: Epithelial membrane antigen
FISH: Fluorescent in situ hybridization
FLAIR: Fluid attenuation inversion recovery
GFAP: Glial fibrillary acidic protein
HCP: Hydrocephalus
IDH: Isocitrate dehydrogenase
LME: Leptomeningeal enhancement
MAP: Microtubule associated protein
MRI: Magnetic resonance imaging
WHO: World Health Organization
